# Development and validation of an early childhood development scale for use in low-resourced settings

**DOI:** 10.1186/s12963-017-0122-8

**Published:** 2017-02-09

**Authors:** Dana Charles McCoy, Christopher R. Sudfeld, David C. Bellinger, Alfa Muhihi, Geofrey Ashery, Taylor E. Weary, Wafaie Fawzi, Günther Fink

**Affiliations:** 1 Harvard T. H. Chan School of Public Health, Boston, MA USA; 2 Harvard Graduate School of Education, Cambridge, MA USA; 3 Harvard Medical School, Boston, MA USA; 4 Ifakara Health Institute, Ifakara, Tanzania

**Keywords:** Early child development, Low-income countries, Measurement, Validation, 0–3

## Abstract

**Background:**

Low-cost, cross-culturally comparable measures of the motor, cognitive, and socioemotional skills of children under 3 years remain scarce. In the present paper, we aim to develop a new caregiver-reported early childhood development (ECD) scale designed to be implemented as part of household surveys in low-resourced settings.

**Methods:**

We evaluate the acceptability, test-retest reliability, internal consistency, and discriminant validity of the new ECD items, subscales, and full scale in a sample of 2481 18- to 36-month-old children from peri-urban and rural Tanzania. We also compare total and subscale scores with performance on the Bayley Scales of Infant Development (BSID-III) in a subsample of 1036 children. Qualitative interviews from 10 mothers and 10 field workers are used to inform quantitative data.

**Results:**

Adequate levels of acceptability and internal consistency were found for the new scale and its motor, cognitive, and socioemotional subscales. Correlations between the new scale and the BSID-III were high (*r* > .50) for the motor and cognitive subscales, but low (*r* < .20) for the socioemotional subscale. The new scale discriminated between children’s skills based on age, stunting status, caregiver-reported disability, and adult stimulation. Test-retest reliability scores were variable among a subset of items tested.

**Conclusions:**

Results of this study provide empirical support from a low-income country setting for the acceptability, reliability, and validity of a new caregiver-reported ECD scale. Additional research is needed to test these and other caregiver reported items in children in the full 0 to 3 year range across multiple cultural and linguistic settings.

## Background

Mounting evidence suggests the importance of investing in early childhood development (ECD) for enhancing the economic, health, and educational status of individuals, communities, and nations [1–4]. Over the past several decades, a number of well-validated tools have been developed for measuring individual children’s motor, cognitive, language, and social functioning during the first years of life (e.g., Griffiths Mental Development Scales, Denver Developmental Screening Test, Bayley Scales of Infant and Toddler Development). These direct assessments are typically done by clinically trained personnel and provide detailed information on individuals’ developmental status that can be used for informing clinical decisions, understanding developmental processes, or testing the efficacy of early interventions [5].

Despite their utility in capturing rich data, individual-level assessments are limited in their ability to provide estimates of population-level developmental status for several reasons. First, many of these assessments are quite costly in terms of their copyrights, the time they take to administer, as well as the resources necessary to train assessors, making them impractical for use at scale [6]. Second, the majority of existing developmental assessments have been created with one particular—primarily high-resourced, Western—cultural context in mind. Although great advances have been made recently in developing new tools for non-Western, low-resourced settings (e.g., the Malawi Developmental Assessment Tool, the Inter-American Development Bank’s PRIDI tool, the Developmental Milestones Checklist, the East Asia and Pacific Early Child Development Scales), the utility of these assessments for making generalizations outside of the context in which they were developed is unknown [7, 8]. Finally, many comprehensive developmental assessments have focused primarily on motor, cognitive, and language development, while neglecting to integrate early manifestations of social, emotional, and regulatory competence. Although many socioemotional skills vary in importance and developmental determinants cross-culturally, research has increasingly shown the early emergence of a core, basic set of these capacities to be strongly related to later-life outcomes in diverse parts of the world [6, 9–13].

In recent years, several new tools have been developed to address these limitations and provide comprehensive population-level data in older children (e.g., UNICEF’s Early Childhood Development Index for 3- and 4-year-olds, the Early Development Index for school-aged children) [14, 15]. Still, no such scale exists for the under-three age period, when children’s brains and bodies are developing most rapidly and are most susceptible to intervention [16]. Given that target 4.2 of the Sustainable Development Goals aims to “ensure that all girls and boys have access to quality ECD,” measuring children’s developmental status at the population level is of important policy relevance [17]. Internationally validated tools would provide a new opportunity for global ECD advocates to quantify children’s needs across countries and regions, to make more informed decisions regarding policies and resource allocation, and to monitor progress in achieving global goals congruent with the post-2015 agenda [18].

In this study, we describe the development of a set of caregiver-reported items for quickly and easily measuring the motor, cognitive, and socioemotional skills of children under three living in low-resourced settings, collectively known as the Caregiver-Reported Early Development Index (CREDI). Our focus on a caregiver report format allows us to address several practical and conceptual challenges of using direct assessment with large groups of infants and toddlers. Compared to direct assessments, caregiver reports require limited training and implementation time, provide a more generalizable perspective on children’s skills and behaviors across time and setting, are more appropriate for capturing socioemotional skills, and are less likely to be biased against children who are unfamiliar with clinical assessments, who are shy with strangers, or who do not understand verbal instructions [5]. In particular, the CREDI is designed to be 1) simple and clear enough to be answered by a caregiver with minimal formal education, 2) short enough to be feasibly integrated within large-sample household data collection efforts, 3) sufficiently “culturally neutral” to allow for cross-context comparison, and 4) adequately aligned with “gold standard” direct assessment measures of proven clinical and developmental utility. In creating the CREDI, our ultimate aim is to generate a new tool that will serve to provide conceptually rich, developmentally informed, population-level data on global progress in alleviating ECD-related inequities and meeting target 4.2 of the SDGs. In the present paper, we detail the initial validation of the CREDI using qualitative and quantitative data among 18- to 36-month-old children in peri-urban and rural Tanzania, including evidence of the individual items’ and overall scale’s acceptability, reliability, and validity. We conclude by describing the implications of this generative work for future validation and expansion efforts.

## Methods

### Study sample

The sample for the present study was comprised of children 18 to 36 months who had previously participated in a neonatal vitamin A supplementation trial in the Morogoro region of Tanzania (registered at anzctr.org.au as ACTRN12610000636055) [19], as well as the person in the household who reported to spend the most time caring for that child (i.e., his or her primary caregiver). This particular area of Tanzania was selected over alternate study locations due to its 1) track record and infrastructure for conducting high-quality early childhood research, and 2) similarity to the broader population of Tanzania with regard to its high prevalence of poverty and malnutrition, mix of peri-urban and rural settings, and cultural diversity. Newborns were eligible for the original vitamin A study if they were able to feed orally, were born within the past 72 h, were not already enrolled in other clinical trials, their family intended to reside in the study area for at least 6 months post-delivery, and their caregivers provided written informed consent. Notably, results of the original vitamin A trial revealed no detectable impacts on children’s developmental outcomes [19], suggesting that randomization in the original study should not have affected the results of the present analysis.

For the original trial, a total of 20,104 randomly selected children living in Morogoro region were enrolled. For the follow-up study, sampling was restricted to children from the original trial living within the Ifakara Demographic Surveillance Site (IHI DSS). No other exclusion criteria (e.g., based on disability or health status) were applied. Given this, the sample is representative of the greater Ifakara area, with all eligible children in Ifakara town and the surrounding villages being equally likely to be selected for participation. In keeping with the aim of the study to validate the CREDI for children 18–36 months, only those within this age range were selected, with the specific age of the child varying non-systematically based on the timing of initial recruitment to the vitamin A study and the timing of the CREDI assessment (38% 18–23mo, 25% 24–29mo, and 38% 30–36mo). Children in the present sample were found to be comparable to those sampled from the 2015–2016 Tanzanian national Demographic Health Survey (DHS) in rates of stunting (43.3% vs. 43.8%, respectively) [20]. Compared to the Tanzanian average, mothers in this sample were more likely to have attended primary school than those in the DHS (87.9% vs. 61.9%, respectively), but less likely to have completed secondary school or higher (7.3% versus 23.4%).

### Ethics

All study protocols were approved by institutional review boards (IRBs) at the Harvard T. H. Chan School of Public Health, the National Institute of Medical Research of Tanzania, and the Ifakara Health Institute. Caregivers provided written consent for their own participation and the participation of their children after a field worker read the consent out loud and answered any questions. All study staff were trained and monitored in IRB-approved procedures for identifying participant needs and, as necessary, providing referrals to local physical and mental health services.

### Item development phase

Multiple steps were taken to develop the ECD items analyzed in this study. First, we reviewed the ECD measurement literature to help us to define 1) the purpose of the scale, 2) the age-appropriate developmental domains and constructs to be covered by the scale, and 3) the validation plan. Second, and based on the literature review, we built an inventory of existing measurement tools from high-, middle-, and low-income country contexts (see Table 5 in Appendix A), and identified gaps in their coverage of our age-specific domains and constructs. Third, we selected, adapted, and/or created an initial set of items based on the following criteria:

Each item must:

1. have evidence for face, construct, and/or criterion validity for representing one of the core ECD domains^1^
2. be developmentally appropriate for children 18 to 36 months^2^
3. be reportable by a primary caregiver on a yes/no response scale (i.e., the item cannot be task-based, cannot be rated on a continuous scale,^3^ and must be sufficiently concrete that a caregiver would already be familiar with the specified behavior/skill in the child)
4. be simple in wording to allow for easy translation and comprehension by caregivers with minimal formal education
5. have the potential to discriminate between individuals (i.e., indicate a high likelihood of variability in response)
6. not be subject to severe social desirability (i.e., a caregiver will not feel compelled to respond in a particular way in order to please the assessor or avoid shame/embarrassment)
7. be culturally neutral (i.e., involve skills, behaviors, objects, ideas, or terminology that are common across contexts)

Each of these three phases was led by the study authors, with results reviewed by a group of advisory team members who represented multiple backgrounds (e.g., research, practice, policy), fields (e.g., health, nutrition, psychology, education), and geographical contexts (e.g., United States, sub-Saharan Africa, Asia, Latin America). Advisory group members provided oral and written feedback on study procedures and materials via bi-monthly conference calls, formal surveys, and informal communications (e.g., emails, one-on-one meetings).

Finally, all items were translated and back-translated to/from Swahili by bilingual Tanzanian and American study staff. Discrepancies in translation were resolved based on the consensus of a committee comprised of CREDI team members, local staff, and bilingual Tanzanian community members.

### Qualitative pilot phase

To provide preliminary feedback on the initial set of items, we conducted a series of “cognitive” (qualitative) interviews in December of 2013 with 10 caregiver-child pairs in and around Ifakara, Tanzania (mean age of children = 28.2 months, range = 20–35 months). A local female research scientist with a master’s degree in human development was recruited based on her previous experience conducting qualitative research in the study community. The interviewer conducted interviews one-on-one with caregivers in children’s homes using a semi-structured interview protocol designed to elaborate each item’s acceptability, clarity, and applicability, as well as the comprehensiveness and redundancy of the scale as a whole [21, 22]. Specifically, the interviewer asked the caregiver (all of whom happened to have been mothers) to respond to each item based on her child’s ability or behavior. The interviewer then asked one or more in a series of seven follow-up questions designed to elicit the caregiver’s perceptions of the item, her thought process in responding to the item, and/or her suggestions for improving the item. At the end of each interview, the caregiver was also asked to give her general impressions of what positive ECD means to her, the acceptability of the scale, and whether she had any suggestions for improving the scale. (For the full interview protocol, contact the first author.) The results of these interviews were used to provide preliminary information regarding the overall acceptability of the scale, as well as to identify items that required further adaptation or elimination prior to larger-scale quantitative testing.

### Quantitative pilot phase

Following the qualitative phase, we conducted a full quantitative pilot from January to October of 2014 in 2481 caregiver-child pairs, of which 2320 (93.5%) included mothers, 68 (2.7%) included fathers, and 93 (3.8%) included other family members (e.g., grandparents, aunts). Child-caregiver pairs who participated in the qualitative pilot portion of the validation study were excluded from participation in the full quantitative pilot phase. Of the 4356 children randomly selected for a home visit, 2481 (57.0%) completed the visit, 558 (12.8%) were temporarily away, 1204 (27.6%) had permanently moved, 60 (1.4%) had died, and 53 (1.2%) had caregivers who refused to participate. The characteristics of those who completed the home visit versus those who were invited but did not complete the home visit are shown in Table 6 in Appendix B and indicate relative similarity across the groups. Each caregiver-child pair was visited in their home, invited and consented to participate, and interviewed using all items on the CREDI. Caregivers also reported on cognitive stimulation using six items from UNICEF’s Multiple Indicator Cluster Survey ECD module capturing adult-child interactions [14] and children’s physical and mental disability using six items from the Ten Questions screener [23]. Stimulation items reflected whether an adult household member had engaged the child in six different activities (e.g., reading, counting, playing, singing) over the preceding 3 days. Children were grouped into low (0–2 activities), moderate (3–4 activities), and high (5–6 activities) stimulation categories for analyses. Disability items reflected children’s difficulty with seeing, hearing, moving, and learning. Children were considered to have a disability if their caregiver answered “yes” to any of the six screening items.

Home visits were completed by eight male, secondary school-educated field workers with previous experience conducting field-based research with families and children in the local area. Field workers were selected based on their performance as data collectors in the original vitamin A study and participated in a 2-day training on the CREDI and other study visit procedures. All workers were also monitored by the study coordinator in the field on a bi-weekly basis to ensure continued adherence to study protocols. During the home visits, field workers rated their perceptions of caregivers’ understanding of and honesty in responding to the CREDI items. They also recorded any questions or concerns stated by the caregivers during the interview. At the end of the visit, field workers measured children’s height to the nearest 0.1 cm. Children less than 24 months were measured using a Seca length board, whereas those 24 months or older were measured using a portable Seca stadiometer. Field workers measured height twice in a row, and if the two values differed by more than 0.2 cm, they repeated the measurement a third time, taking an average of the two closest values. Table 1 shows descriptive statistics for this sample.

**Table 1:** Descriptive characteristics of the quantitative pilot sample

	N	Mean/%	SD	Min	Max
*CREDI* ^a^					
Total score (*n* = 44 items)	2481	0.64	0.17	0.07	0.98
Motor (*n* = 5 items)	2481	0.63	0.24	0.00	1.00
Cognitive (*n* = 19 items)	2481	0.64	0.29	0.00	1.00
Socioemotional (*n* = 20 items)	2481	0.64	0.15	0.10	1.00
*Bayley Scales of Infant Development-III*					
BSID Cognitive	959	60.50	8.67	30	81
BSID Receptive Communication	950	25.78	7.00	5	42
BSID Expressive Communication	947	30.06	8.58	3	46
BSID Fine Motor	955	40.56	6.55	12	62
BSID Gross Motor	960	57.00	5.56	34	70
BSID BOI—Caregiver	1033	1.51	0.35	0	2
BSID BOI—Assessor	1033	1.56	0.25	0	2
*Child and family characteristics*					
Child female	2481	45.6%			
Child age (months)	2481	27.07	6.08	17.03	37.08
Child height-for-age z-score	2177	−1.82	1.28	−5.99	4.94
Child stunted (HAZ < −2)	2177	43.3%			
Child any disability	2481	1.9%			
Proportion of stimulation activities conducted (out of 6)	2480	0.49	0.16	0.00	1.00
Maternal educ—No school	2481	4.6%			
Maternal educ—Primary school	2481	86.2%			
Maternal educ—Secondary school	2481	7.3%			

^a^CREDI mean scores represent proportion of correct responses on the scale or sub-scale. Scores calculated based on the final set of 44 items only

Approximately 60% of caregiver-child pairs were selected by a computer-generated random number draw before their home visit to be invited to an additional clinic visit, which occurred 1 to 6 days after the home visit. Of the 1478 children randomly selected for a clinic visit, 1037 (70.2%) completed the visit, 224 (15.2%) agreed to the visit but did not show up, 57 (3.9%) refused the visit, and the remainder (10.8%) were not scheduled due to logistical reasons (e.g., caregiver or child was ill, no clinic appointments were available). The characteristics of home visit participants who completed the clinic visit versus those who did not complete the clinic visit are shown in Table 7 in Appendix C and indicate relative similarity across the groups. During the clinic visit, a female nurse with training in child development and research re-administered a subset of 11 CREDI items (selected for their conceptual diversity) and conducted an adapted and translated version of the Bayley Scales of Infant Development (BSID-III) [24] with the child, including all direct assessment subscales as well as the Behavior Observation Inventory (BOI). The BSID-III was chosen as the comparison metric for the present study due to its acceptance as a “gold standard” clinical assessment with strong reliability and validity, its complementary direct assessment format, and its previous use by our team in Tanzanian ECD research [25–27].

Because the BSID-III was originally developed in the United States, field and research staff completed a detailed adaptation process over a period of several weeks to improve its applicability within the Tanzanian context. Details of the training, adaptation, and psychometric properties of the BSID-III can be found in Sudfeld et al. [28]. Briefly, six nurses were trained to administer the BSID-III by two American PhD-level psychologists over a 3-week period, after which four nurses were selected as study staff based on quantitative ratings of their performance and knowledge. Study nurses were each monitored by the local study coordinator on a biweekly basis to ensure quality and to avoid assessor drift. To enhance cultural applicability, unfamiliar images and terminology within 13% of BSID-III items (*n* = 30) were replaced using more culturally relevant stimuli (e.g., changing a picture of an apple to a banana) based on local expert consensus. To maintain functional equivalence, replacement stimuli were selected to be of similar size, style, and complexity to original stimuli. Raw scores were used for analyses due to lack of Tanzania-specific age-norms. At the end of the clinic visit, nurses recorded mothers’ questions and any problems that may have precluded full completion of the visit (e.g., child was sick or uncooperative).

Data from the quantitative pilot phase were used at the item level to understand individual items’ distributional properties, including pass/fail rates and levels of non-response (i.e., “don’t know” answers). Test-retest reliability was assessed for the 11 CREDI items tested in both the home and clinic visit. Additional tests of reliability and validity were performed for items that were identified to have sufficient variability (i.e., that did not show evidence for floor or ceiling effects). Specifically, internal consistency was captured within each of the three CREDI domains/subscales using Cronbach’s alpha. Discriminant validity was assessed by comparing CREDI total and subscale scores across a set of child and family characteristics, including child age, gender, stunting status (height-for-age z-score of <2SDs below the WHO standard) [29], caregiver-reported disability, caregiver-reported cognitive stimulation in the home, and maternal education (which was collected at children’s births as part of the original vitamin A study). Finally, concurrent validity was assessed by correlating each CREDI subscale score with the corresponding BSID-III raw score. Psychological field standards (e.g., Cicchetti, [30]) were used as the basis for determining acceptability of the items’ and subscales’ reliability and validity.

### Field staff interviews

At the end of the quantitative pilot phase, 10 qualitative “exit” interviews were conducted with field staff (including six field workers, three nurses, and one field supervisor) to identify areas of confusion, difficulty, or lack of clarity in the CREDI based on their experiences over 9 months of data collection.

## Results

### Item development & qualitative interviews

Review of the literature and consultation with ECD experts resulted in the identification of three primary domains—motor, cognitive/language, and socioemotional skills—and 12 constructs or subdomains for inclusion in the CREDI (see Table 2). Based on a review of existing ECD measurement tools (see Table 5 in Appendix A) and the process of identifying conceptual gaps, an initial set of items was developed by the core research team. Whereas many of these items were highly similar to questions from existing ECD assessments, a substantial number—particularly from the socioemotional domain, where the largest conceptual gaps were identified—were completely novel. Following a round of revisions to the items by the ECD expert team, a total of 92 items were submitted for initial qualitative pilot testing. Following qualitative interviews, 22 items (*n* = 7 from motor, *n* = 8 from cognitive, and *n* = 7 from socioemotional) were dropped from the CREDI for the following reasons: the item was too easy/hard for children of this age group (*n* = 10), the item was redundant with another item (*n* = 8), the item was confusing and could not be easily clarified (*n* = 3), and the item was culturally inappropriate and could not be easily adapted (*n* = 1). Of the remaining 70 items, 15 (*n* = 1 for motor, *n* = 7 for cognitive, and *n* = 7 for socioemotional) were adapted prior to the quantitative pilot based on suggestions from cognitive interview participants and additional consultation with local experts. These adaptations primarily involved the addition of examples to improve item clarity, such as changing “Does the child know any numbers?” to “Does the child know any numbers (e.g., one, two, three)?” In several instances, words relating to culturally specific objects (e.g., toys) were removed or replaced.

**Table 2:** Domains and constructs of the CREDI

DOMAINS	Motor	Cognitive	Socioemotional
CONSTRUCTS	1) Fine 2) Gross	1) Expressive language 2) Receptive language 3) Preacademic skills/knowledge 4) Reasoning & problem solving	1) Early executive function & effortful control 2) Emotion regulation 3) Externalizing symptoms 4) Internalizing symptoms 5) Reactivity & soothability 6) Social competence

### Acceptability

Cognitive interviews revealed that 10/10 caregivers were cooperative with and felt pleased by the items, and 9/10 felt that “there were no right or wrong answers.” (One mother of a 20-month-old child reported, “I was uncomfortable when you asked me things which my child cannot do, as she is too young.”) Field workers’ average ratings of whether the caregivers understood the questions during the quantitative pilot was 3.85 (SD = 0.28) and whether they appeared to answer truthfully was 3.77 (SD = 0.36) on a scale of 1 (No, not at all) to 4 (Yes, all questions). In addition, exit interviews of field staff identified no problems with items’ demand characteristics, with the exception of a socioemotional item capturing whether the child “gets along well with other children most of the time” that was reported by five of the 11 field workers as eliciting problems with social desirability.

### Item analysis

**Fig. 1:**
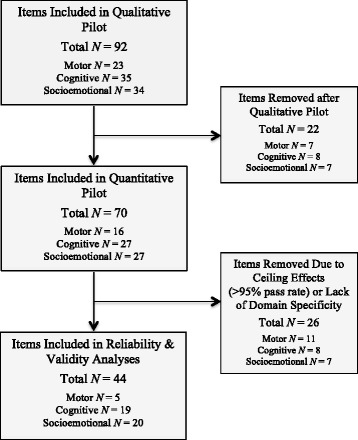
Item selection tree

**Fig. 2:**
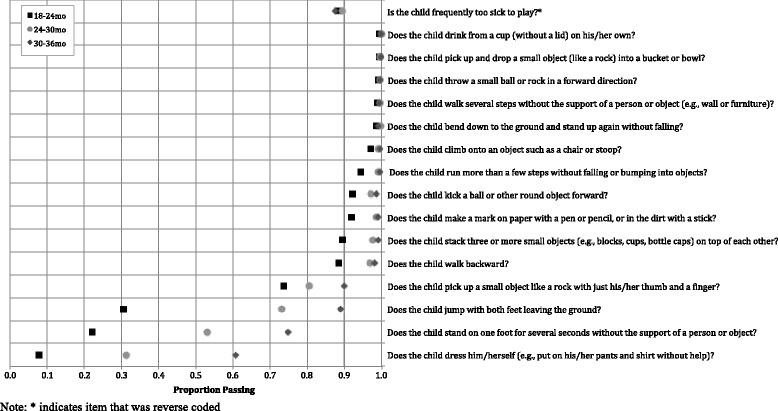
Proportion children passing each motor item, by age (*n* = 2481)

**Fig. 3:**
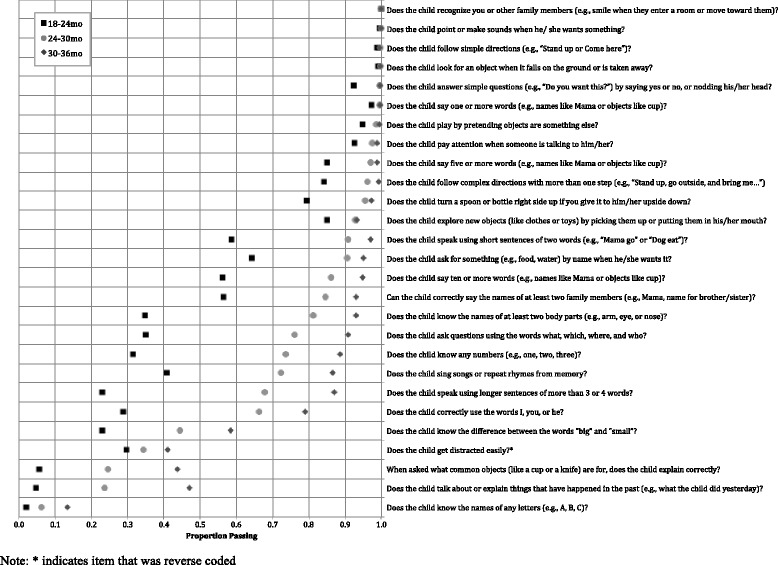
Proportion children passing each cognitive item, by age (*n* = 2481)

**Fig. 4:**
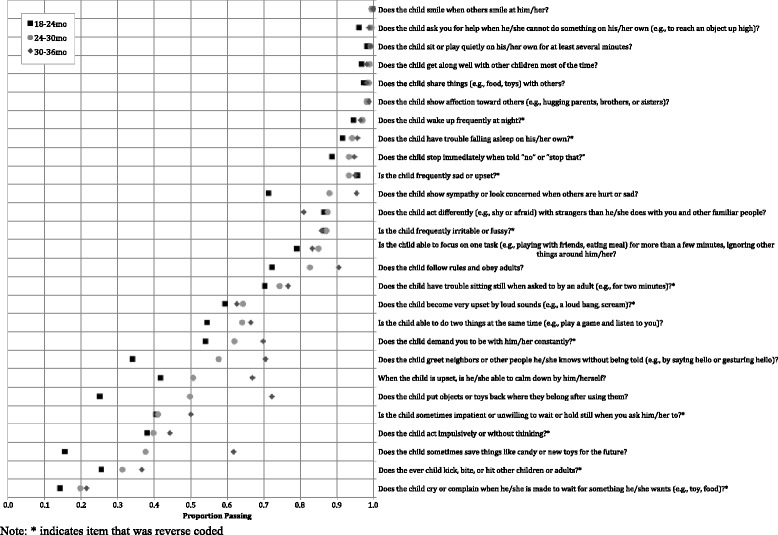
Proportion children passing each socioemotional item, by age (*n* = 2481)

Results of item analyses to understand the completeness, distribution, and relative difficulty of each item as measured during the quantitative pilot home visit can be found in Table 8 in Appendix D. Results revealed that 25 of the 70 items (*n* = 10 for motor, *n* = 8 for cognitive, *n* = 7 for socioemotional) showed evidence of ceiling effects, with pass rates of >95%. In general, these items tended to represent more basic developmental skills that may be more appropriate for children <18 months (e.g., walking, achieving object permanence, saying one word, showing affection). These items were removed from the final subscales used for reliability and validity analyses. Figure 1 summarizes the item selection process. Figures 2, 3 and 4 show score distributions by age.

“Don’t know” responses were infrequent across the CREDI, with an average of 1.8% of the sample responding “don’t know” for any given item during the home visit. In comparison, among 1037 BSID-III assessments, 9.9% were incomplete and an additional 10.1% were flagged by nurses as challenging or unreliable due to children’s illness, injury, uncooperativeness, or distraction. Of the items that were most frequently answered as “don’t know,” the majority were also acknowledged as unclear in the qualitative interviews due translation difficulties (e.g., inability to find an equivalent word or set of words for “distracted” in Swahili) or lack of a concrete behavioral marker (e.g., ambiguity of what it means to show sympathy or concern).

### Reliability

A total of 26 items were excluded from the original 70-item set due to ceiling effects (*n* = 25) and the lack of conceptual fit with a specific developmental domain (*n* = 1, “too sick to play”). Cronbach’s alpha coefficients calculated in the final set of 44 items suggested acceptable internal consistency/inter-item reliability for motor (α = .68), cognitive (α = .90), and socioemotional (α = .68) items. Kappa coefficients were used to capture the reliability of responses from the same caregiver to 11 items administered at both the home and clinic visits (see Table 3). It should be highlighted that the Kappa statistic was originally developed as a measure of inter-rater reliability, where two raters directly observe or assess the same individual at the same time. In the case of the present study, our Kappas capture both test-retest reliability (with an average time between study visits of 3.17 days [SD = 2.11]) and inter-rater reliability (between male home visitors and female clinic nurses). Given this, they represent both true variation in children’s skills over time, as well as multiple potential sources of measurement error. As such, we might expect our Kappas to be lower than those used simply to capture inter-rater reliability. Indeed, results indicate differential reliability, with two items showing moderate reliability (Kappa ≥ 0.40), six items showing fair reliability (Kappa ≥ 0.20), 2 items showing slight reliability (Kappa ≥ 0.00), and one item showing poor reliability (Kappa < 0.00). Additional analyses revealed no consistent evidence for systematic differences in mean scores across home and clinic visits (see Table 3) or for substantial differences in Kappa values based on the time delay between the home and clinic visit (contact first author for detailed results).

**Table 3:** Test-retest reliability of 11 select items across data collection contexts with same caregiver reporter (*n* = 962)

	Mean score		Difference in means	% Agreement	Kappa
	Home interview	Clinic interview			
Does the child walk several steps without the support of a person or object (e.g., wall or furniture)?	0.99	0.99	0.00	99.3%	.663
Does the child know the names of at least two body parts (e.g., arm, eye, or nose)?	0.67	0.65	0.02	80.4%	0.563
Does the child say five or more words (e.g., names like Mama or objects like cup)?	0.91	0.93	−0.02	90.4%	0.335
When asked what common objects (like a cup or a knife) are for, does the child explain correctly?	0.34	0.20	0.14	74.4%	0.363
Does the ever child kick, bite, or hit other children or adults?^a^	0.45	0.31	0.14	68.0%	0.333
Does the child pick up a small object like a rock with just his/her thumb and a finger?	0.78	0.84	−0.06	78.0%	0.276
Does the child get along well with other children most of the time?	0.92	0.97	−0.05	92.2%	0.241
When the child is upset, is he/she able to calm down by him/herself?	0.45	0.59	−0.14	60.0%	0.213
Does the child pay attention when someone is talking to him/her?	0.90	0.95	−0.05	87.8%	0.156
Does the child follow simple directions (e.g., “Stand up or Come here”)?	0.98	0.99	−0.01	97.7%	0.144
Is the child sometimes impatient or unwilling to wait or hold still when you ask him/her to?^a^	0.38	0.48	−0.10	49.4%	−0.020
*Average*	*0.71*	*0.72*	*−0.01*	*79.8%*	*0.297*

CREDI mean scores represent proportion of correct responses on the item

^a^Indicates item that was reverse coded

### Validity

Table 4 shows the results of tests of discriminant validity for CREDI scores based on child and family characteristics. These results show significantly higher total CREDI scores for children who were older, non-stunted, non-disabled, and from high-stimulation households at the time of data collection. Effect sizes for these differences ranged from small (*d* ≈ 0.20SD) for stunting, to large (d > 0.50SD) for age, disability, and stimulation. No significant (*p* < .05) differences were observed for CREDI scores across gender or maternal education with the exception of socioemotional scores, which were highest for children of non-educated mothers (*d* ≈ 0.20SD).

**Table 4:** CREDI mean scores (SE) by subgroup (*n* = 2481)

	Total	Motor	Cognitive	Socioemotional
*Child age*				
18–24 mo^a^ (*n* = 934)	0.50 (0.005)	0.45 (0.008)	0.44 (0.007)	0.57 (0.005)
> 24–30 mo^b^ (*n* = 614)	0.67 (0.005)	0.67 (0.010)	0.69 (0.008)	0.65 (0.005)
> 30–36 mo^c^ (*n* = 933)	0.76 (0.004)	0.82 (0.007)	0.79 (0.005)	0.71 (0.004)
* dif*	*F*(2, 2478) = 965.00** a < b: *d* = 1.00** b < c: *d* = 0.51** a < c: *d* = 1.51**	*F*(2, 2478) = 560.63** a < b: *d* = 0.78** b < c: *d* = 0.50** a < c: *d* = 1.28**	*F*(2, 2478) = 860.93** a < b: *d* = 1.03** b < c: *d* = 0.41** a < c: *d* = 1.44**	*F*(2, 2478) = 234.26** a < b: *d* = 0.52** b < c: *d* = 0.39** a < c: *d* = 0.92**
*Child gender*				
Male (*n* = 1349)	0.64 (0.005)	0.64 (0.008)	0.63 (0.007)	0.64 (0.004)
Female (*n* = 1132)	0.64 (0.005)	0.64 (0.009)	0.64 (0.007)	0.64 (0.004)
*dif*	*t*(2479) = −0.70 *d* = 0.03	*t*(2479) = 0.08 *d* = −0.00	*t*(2479) = −1.34 *d* = 0.06	*t*(2479) = 0.31 *d* = −0.01
*Child stunting*				
Non-stunted (*n* = 1222)	0.66 (0.005)	0.66 (0.008)	0.66 (0.007)	0.65 (0.004)
Stunted (*n* = 955)	0.62 (0.006)	0.62 (0.010)	0.60 (0.008)	0.65 (0.005)
*dif*	*t*(2175) = 4.79** *d* = −0.19**	*t*(2175) = 3.46** *d* = −0.13**	*t*(2175) = 6.09** *d* = −0.25**	*t*(2175) = 0.86 *d* = −0.04
*Child disability*				
No disability (*n* = 2434)	0.64 (0.003)	0.65 (0.006)	0.64 (0.005)	0.64 (0.003)
Any disability (*n* = 47)	0.53 (0.032)	0.45 (0.050)	0.48 (0.047)	0.60 (0.025)
*dif*	*t*(2479) = 4.43** *d* = −0.65**	*t*(2479) = 4.73** *d* = −0.69**	*t*(2479) = 4.45** *d* = −0.65**	*t*(2479) = 1.81+ *d* = −0.27+
*Stimulation*				
Low stimulation^a^ (*n* = 847)	0.63 (0.005)	0.62 (0.10)	0.63 (0.008)	0.64 (0.005)
Mod stimulation^b^ (*n* = 1498)	0.63 (0.005)	0.65 (0.08)	0.62 (0.006)	0.64 (0.004)
High stimulation^c^ (*n* = 135)	0.75 (0.011)	0.77 (0.021)	0.81 (0.014)	0.68 (0.014)
*dif*	*F*(2, 2477) = 29.57** a < b: *d* = 0.02 b < c: *d* = 0.67** a < c: *d* = 0.68**	*F*(2, 2477) = 17.41** a < b: *d* = 0.09+ b < c: *d* = 0.45** a < c: *d* = 0.54**	*F*(2, 2477) = 41.00** a < b: *d* = −0.02 b < c: *d* = 0.80** a < c: *d* = 0.77**	*F*(2, 2477) = 3.65* a < b: *d* = 0.03 b < c: *d* = 0.22* a < c: *d* = 0.25*
*Maternal education*				
No education^a^ (*n* = 113)	0.63 (0.016)	0.61 (0.029)	0.61 (0.022)	0.67 (0.015)
Primary school^b^ (*n* = 2138)	0.64 (0.004)	0.65 (0.026)	0.64 (0.005)	0.65 (0.003)
Secondary school^c^ (*n* = 181)	0.61 (0.013)	0.63 (0.022)	0.61 (0.019)	0.62 (0.012)
*dif*	*F*(2, 2429) = 2.63+ a < b: *d* = 0.14 b < c: *d* = −0.18+ a < c: *d* = −0.04	*F*(2, 2429) = 1.36 a < b: *d* = 0.20 b < c: *d* = −0.08 a < c: *d* = 0.12	*F*(2, 2429) = 2.29+ a < b: *d* = 0.19 b < c: *d* = −0.14 a < c: *d* = 0.05	*F*(2, 2429) = 4.36** a < b: *d* = −0.04 b < c: *d* = −0.19* a < c: *d* = −0.24*

**p < .01, *p < .05, +*p* < .10; CREDI mean scores represent proportion of correct responses on the scale or sub-scale; *d* indicates effect size of standardized mean differences as represented by Cohen’s *d*

**Fig. 5:**
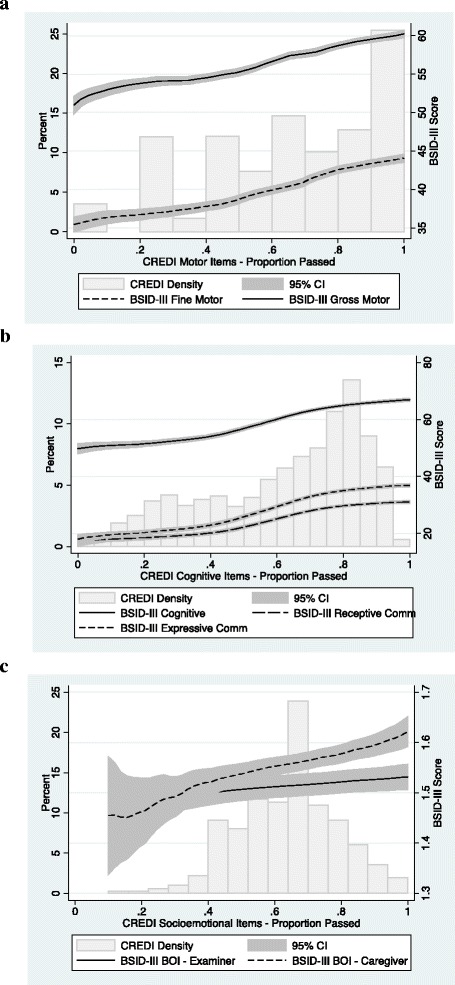
Histogram of CREDI distribution and local polynomial graph of the relation between CREDI and BSID-III subscale scores (line w/95% CI): Motor (Panel **a**), Cognitive (Panel **b**), & Socioemotional (Panel **c**)

Figure 5 shows the correlations between the CREDI and BSID-III subscales. Linear bivariate correlations between the CREDI motor items and the BSID-III fine and gross motor subscales were *r* = .50 and *r* = .51, respectively. Correlations between the CREDI cognitive items and the BSID-III cognitive, receptive communication, and expressive communication subscales were *r* = .68, *r* = .69, and *r* = .73, respectively. All of these correlations were significant at the *p* < .001 level. Correlations between the CREDI socioemotional items and the BSID-III BOI were much smaller, at *r* = .16 (*p* < .001) for the caregiver-reported BOI and *r* = .09 (*p* < .01) for the examiner-reported BOI.

## Discussion

The primary aim of the present study was to describe initial evidence for the acceptability, reliability, and validity of the newly developed CREDI as a measure of ECD designed for feasible use within standard household surveys in low-resourced settings. Results of our initial validation effort in Tanzania suggest that the CREDI tool may provide a valid method for capturing young children’s development across motor, cognitive, and socioemotional domains. In particular, the CREDI was able to clearly discriminate between the skills of younger versus older children, children with adequate versus low nutritional status, disabled versus non-disabled children, and children from more versus less cognitively stimulating households, while showing evidence for equality across gender and maternal education within a large quantitative sample. Collectively, the items also showed adequate criterion validity with the BSID-III motor, cognitive, and communication subscales, which are “gold standard” direct assessments of children’s early developmental status often used in clinical settings by highly trained staff.

In addition to showing positive evidence for validity, the CREDI was found to be an acceptable tool for use in low-resourced settings. It was well understood by respondents and quick to implement (taking an average of 20 min to administer in total) by trained field staff with the equivalent of a secondary education. Furthermore, initial findings suggest that the caregiver report format may be advantageous for use with young children in low-resourced settings in order to avoid problems with non-compliance (e.g., due to unfamiliarity with testing situations, fear of unfamiliar adults, child illness, etc.) that were found to affect the quality and completeness of nearly 20% of BSID-III direct assessments.

Although the CREDI as a whole shows promise as an acceptable and valid measurement tool, test-retest reliability was low for many individual items, and particularly for those that qualitative interview respondents noted were difficult to translate or lacking in examples, benchmarks, or behavioral markers. Given that no systematic differences were found based on the interviewer, setting, or time between visits, these results suggest that further adaptation is needed to make items as concrete as possible and reduce respondent “guessing.” Additional reliability testing, qualitative work, and empirical analysis (e.g., item response theory) are also warranted in future work to ensure that items’ interpretation is occurring similarly across time, context, respondent, and assessor.

In addition, these results revealed a relatively weak correspondence between the socioemotional items and the BSID-III BOI. This low correlation was not particularly surprising given that 1) the BOI was not designed as a measure of socioemotional functioning, per se, and 2) our aim in developing the socioemotional items was to capture a large breadth of important but potentially non-overlapping developmental constructs. Our review of the literature and consultation with ECD experts revealed that the vast majority of previous measurement tools (like the BSID-III) have focused on young children’s motor and cognitive development, with far fewer options for capturing social, emotional, and higher-order cognitive processes like self-regulation and executive function that are increasingly being shown by the literature to predict later life outcomes [10, 12, 31–33]. Given that our socioemotional items showed adequate reliability and validity in other ways (e.g., internal consistency, discrimination by age, caregiver-reported stimulation, etc.), we are confident that their inclusion represents an important advance over previous work in this age group. At the same time, we acknowledge the need for further validation against alternative socioemotional measurement approaches (e.g., the Ages and Stages personal-social and socio-emotional scales, observer ratings of child behavior during assessment) and clinical diagnoses, as well as examinations of predictive validity over time in diverse settings, particularly given a lack of understanding of these early skills cross-culturally. Additional research is also needed to explore the somewhat counterintuitive finding that less educated caregivers report the highest levels of socioemotional development for their children.

Despite the strengths of this study, the research presented also has several important limitations that must be addressed through future work. First, and most importantly, our focus on a single geographic context substantially limits the generalizability of these results. Second, the number of qualitative interviews conducted in this study was quite small, and focused only on mothers. Third, as is noted previously, our lack of a “gold standard” metric against which to compare our socioemotional items limits our understanding of their concurrent validity. Fourth, our additional measures of context and disability were limited and coarse, and may not have been suitable for fully describing the risks and challenges faced by children. Finally, the cross-sectional nature of our data collection effort precludes our ability to draw conclusions about the CREDI’s long-term predictive validity. To address these limitations, we plan to continue validation of the CREDI using 1) a large number of geographically, linguistically, and culturally diverse contexts, 2) different types of caregivers, 3) a wider range of locally-generated comparison and diagnostic metrics, and 4) longitudinal data. In particular, additional qualitative and quantitative work is currently underway in multiple countries to improve the clarity and objectivity of items in an attempt to improve test-retest reliability. Based upon the results of these ongoing and future efforts, we hope to finalize and disseminate the CREDI as an open-source tool for governments, agencies, and organizations to quantify developmental status at a population level and track progress in alleviating ECD-related disparities around the world.

## Conclusions

Given growing justification for and investment in the promotion of positive development in the first 1000 days of life, providing a tool for quantifying and monitoring early developmental outcomes—particularly for the 89% of children under five globally who live in low- and middle-income country contexts—is critically important [34]. Designed as a comprehensive, caregiver-reported assessment of ECD for children under three, the aim of the CREDI is to provide low-cost, large-scale data that will facilitate decision making regarding intervention and resource allocation, and track global progress in alleviating early developmental disparities. The results of the present study suggest that overall, the CREDI worked well for capturing ECD behaviors and skills in 18- to 36-month-old children within Tanzania. Additional research in diverse linguistic and cultural contexts and younger age groups is needed to ensure the CREDI’s utility prior to full dissemination.

## Appendix A

**Table 5:** List of measurement tools consulted in preparing the CREDI items

Name	Citation
*Developed in high-income countries*	
Ages & Stages Questionnaire	Bricker, Diane D., Jane Squires, and Linda Mounts. *Ages & stages questionnaires: A parent-completed, child-monitoring system*. Baltimore (MD): Paul H. Brookes, 1999.
Bayley Scales of Infant and Toddler Development—III	Bayley, Nancy. *Bayley Scales of Infant and Toddler Development: Bayley-III*. Harcourt Assessment, Psych. Corporation, 2006.
Behavior Rating Inventory of Executive Function	Gioia, G. A., Isquith, P. K., Guy, S. C., & Kenworthy, L. (2000). Test review Behavior Rating Inventory of Executive Function. *Child Neuropsychology*, *6*(3), 235–238.
Child Behavior Checklist	Achenbach, Thomas M., and C. Edelbrock. *Child Behavior Checklist. Burlington (Vt)* 7 (1991).
Minnesota Child Development Inventory	Ireton, Harry, and Edward Thwing. *Minnesota Child Development Inventory*. Minneapolis: Behavior Science Systems, 1974.
Denver Developmental Screening Test	Frankenburg, William K., and Josiah B. Dodds. The Denver developmental screening test. *The Journal of Pediatrics* 71.2 (1967): 181–191.
Early Childhood Longitudinal Study—Birth Cohort, 9 and 24mo parent report	Andreassen, Carol, and Philip Fletcher. “Early Childhood Longitudinal Study, Birth Cohort (ECLS-B): Psychometric Report for the 2-Year Data Collection. Methodology Report. NCES 2007-084.” *National Center for Education Statistics* (2007).
Home Observation for Measurement of the Environment (HOME)	Caldwell, Bettye M., and Robert H. Bradley. *Home Observation for Measurement of the Environment*. Little Rock: University of Arkansas at Little Rock, 1984.
Infant Behavior Questionnaire	Gartstein, Maria A., and Mary K. Rothbart. “Studying infant temperament via the revised Infant Behavior Questionnaire.” *Infant Behavior and Development* 26.1 (2003): 64–86.
Kaufman Assessment Battery for Children	Kaufman, Alan S. *K-ABC: Kaufman Assessment Battery for Children: Interpretive manual*. Circle Pines, MN: American Guidance Service, 1983.
MacArthur-Bates Communicative Development Inventory	Fenson, Larry, et al. *MacArthur-Bates Communicative Development Inventories*. 2007.
Parents’ Evaluation of Developmental Status	Glascoe, Frances Page. *Collaborating with parents: Using Parents’ Evaluation of Developmental Status to detect and address developmental and behavioral problems*. Ellsworth & Vandermeer Press, 1998.
Leiter International Performance Scale	Roid, G., & Miller, L. (1997). Leiter International Performance Scale—Revised. Wood Dale, IL: Stoelting
Strengths & Difficulties Questionnaire	Goodman, Robert. “The Strengths and Difficulties Questionnaire: A research note.” *Journal of Child Psychology and Psychiatry* 38.5 (1997): 581–586.
Vineland Adaptive Behavior Questionnaire	Sparrow, Sara S., David A. Balla, and Domenic V. Cicchetti. *Vineland Adaptive Behavior Scales: Interview edition, survey form manual*. Circle Pines, MN: American Guidance Service, 1984.
*Developed in low- and middle-income countries*	
Kilifi Developmental Inventory	Abubakar, A., et al. “Monitoring psychomotor development in a resource limited setting: an evaluation of the Kilifi Developmental Inventory.” *Annals of Tropical Paediatrics: International Child Health* 28.3 (2008): 217–226.
Malawi Developmental Assessment Tool	Gladstone, Melissa, et al. “The Malawi Developmental Assessment Tool (MDAT): the creation, validation, and reliability of a tool to assess child development in rural African settings.” *PLoS medicine* 7.5 (2010): e1000273.
Early Childhood Development Index	UNICEF. *Multiple Indicator Cluster Surveys (MICS)*, 2009–2012.
Rapid Neurodevelopmental Assessment Instrument	Khan, Naila Zaman, et al. “Validation of Rapid Neurodevelopmental Assessment Instrument for under-two-year-old children in Bangladesh.” *Pediatrics* 125.4 (2010): e755–e762. Khan, Naila Z., et al. “Validation of Rapid Neurodevelopmental Assessment for 2-to 5-year-old children in Bangladesh.” *Pediatrics* 131.2 (2013): e486–e494.
World Health Organization Gross Motor Milestones	Onis, Mercedes. “WHO Motor Development Study: Windows of achievement for six gross motor development milestones.” *Acta Paediatrica* 95.S450 (2006): 86–95.

## Appendix B

**Table 6:** Characteristics of children selected for participation in the CREDI study (*n* = 4356) stratified by those who completed the home visit (*n* = 2481) versus selected participants who did not complete the home visit (*n* = 1875)

	Completed home visit Mean ± SD or frequency (%)	Did not complete home visit Mean ± SD or frequency (%)
*Family characteristics*		
Maternal age at birth (years)	26.3 ± 6.9	25.8 ± 7.1
Maternal education		
No school	113 (4.6)	70 (3.7)
Primary school	2138 (86.2)	1558 (83.1)
Secondary plus	181 (7.3)	217 (11.6)
Wealth Quintile		
Q1 (Poorest)	301 (12.5)	236 (12.6)
Q2	414 (16.7)	263 (14.0)
Q3	371 (15.0)	207 (11.0)
Q4	538 (21.7)	383 (20.4)
Q5 (Richest)	763 (30.8)	722 (38.5)
*Child Characteristics*		
Female	1131 (45.6)	859 (45.8)
Firstborn	682 (27.5)	625 (33.3)
Low birth weight (<2500 g)	411 (16.6)	359 (19.1)
Preterm (<37 weeks)	130 (15.3)	181 (15.0)
Improved water	2364 (95.3)	1777 (94.8)
Flush toilet	628 (25.3)	547 (29.2)

## Appendix C

**Table 7:** Characteristics of children who completed the CREDI home visit (*n* = 2481) stratified by those who completed the clinic visit (*n* = 1036) versus those who did not complete the clinic visit (*n* = 1445)

	Completed clinic visit Mean ± SD or frequency (%)	Did not complete clinic visit Mean ± SD or frequency (%)
*Family characteristics*		
Maternal age at birth (years)	26.3 ± 7.2	26.0 ± 7.2
Maternal education		
No school	53 (5.2)	60 (4.2)
Primary school	888 (85.7)	1250 (86.5)
Secondary plus	72 (7.1)	109 (7.5)
Wealth Quintile		
Q1 (Poorest)	142 (13.7)	159 (11.0)
Q2	182 (17.6)	232 (16.1)
Q3	152 (14.6)	219 (15.2)
Q4	229 (22.1)	309 (21.4)
Q5 (Richest)	297 (28.7)	466 (32.2)
*Child characteristics*		
Female	492 (47.5)	639 (44.2)
Firstborn	276 (30.0)	406 (28.1)
Low birth weight (<2500 g)	176 (17.0)	235 (16.3)
Preterm (<37 weeks)	95 (17.8)	102 (13.6)
Improved water	971 (93.9)	1393 (96.4)
Flush toilet	262 (25.3)	366 (25.3)

## Appendix D

**Table 8:** Item-level summary of pass rates and missing data

Motor items	% Don’t know	Proportion passing
Is the child frequently too sick to play? ^b^	0.04%	0.88
Does the child drink from a cup (without a lid) on his/her own?	0.00%	1.00
Does the child pick up and drop a small object (like a rock) into a bucket or bowl?	0.08%	1.00
Does the child throw a small ball or rock in a forward direction?	0.12%	0.99
Does the child walk several steps without the support of a person or object (e.g., wall or furniture)?	0.00%	0.99
Does the child bend down to the ground and stand up again without falling?	0.20%	0.99
Does the child climb onto an object such as a chair or stoop?	0.00%	0.99
Does the child run more than a few steps without falling or bumping into objects?	0.04%	0.98
Does the child kick a ball or other round object forward?	0.89%	0.96
Does the child make a mark on paper with a pen or pencil, or in the dirt with a stick?	0.93%	0.96
Does the child stack three or more small objects (e.g., blocks, cups, bottle caps) on top of each other?	5.48%	0.95
^a^Does the child walk backward?	6.53%	0.94
^a^Does the child pick up a small object like a rock with just his/her thumb and a finger?	10.72%	0.81
^a^Does the child jump with both feet leaving the ground?	8.14%	0.63
^a^Does the child stand on one foot for several seconds without the support of a person or object?	26.40%	0.50
^a^Does the child dress him/herself (e.g., put on his/her pants and shirt without help)?	0.16%	0.34
Cognitive items		
Does the child recognize you or other family members (e.g., smile when they enter a room or move toward them)?	0.00%	1.00
Does the child point or make sounds when he/she wants something?	0.00%	1.00
Does the child follow simple directions (e.g., “Stand up or Come here”)?	0.00%	0.99
Does the child look for an object when it falls on the ground or is taken away?	0.52%	0.99
Does the child say one or more words (e.g., names like Mama or objects like cup)?	0.04%	0.99
Does the child answer simple questions (e.g., “Do you want this?”) by saying yes or no, or nodding his/her head?	0.12%	0.97
Does the child play by pretending objects are something else?	0.08%	0.97
Does the child pay attention when someone is talking to him/her?	0.12%	0.96
^a^Does the child say five or more words (e.g., names like Mama or objects like cup)?	0.00%	0.93
^a^Does the child follow complex directions with more than one step (e.g., “Stand up, go outside, and bring me…”)?	0.04%	0.93
^a^Does the child turn a spoon or bottle right side up if you give it to him/her upside down?	1.05%	0.90
^a^Does the child explore new objects (like clothes or toys) by picking them up or putting them in his/her mouth?	0.28%	0.90
^a^Does the child ask for something (e.g., food, water) by name when he/she wants it?	0.04%	0.82
^a^Does the child speak using short sentences of two words (e.g., “Mama go” or “Dog eat”)?	0.12%	0.81
^a^Does the child say ten or more words (e.g., names like Mama or objects like cup)?	0.12%	0.78
^a^Can the child correctly say the names of at least two family members (e.g., Mama, name for brother/sister)?	0.04%	0.77
^a^Does the child know the names of at least two body parts (e.g., arm, eye, or nose)?	2.58%	0.68
^a^Does the child ask questions using the words what, which, where, and who?	0.60%	0.66
^a^Does the child sing songs or repeat rhymes from memory?	4.19%	0.66
^a^Does the child know any numbers (e.g., one, two, three)?	0.60%	0.63
^aa^Does the child speak using longer sentences of more than 3 or 4 words?	0.08%	0.58
^a^Does the child correctly use the words I, you, or he?	0.81%	0.57
^a^Does the child know the difference between the words “big” and “small”?	3.26%	0.42
^a^Does the child get distracted easily? ^b^	9.27%	0.35
^a^Does the child talk about or explain things that have happened in the past (e.g., what the child did yesterday)?	0.89%	0.25
^a^When asked what common objects (like a cup or a knife) are for, does the child explain correctly?	2.30%	0.24
^a^Does the child know the names of any letters (e.g., A, B, C)?	1.13%	0.07
Socioemotional items		
Does the child smile when others smile at him/her?	0.00%	1.00
Does the child sit or play quietly on his/her own for at least several minutes?	0.00%	0.99
Does the child ask you for help when he/she cannot do something on his/her own (e.g., to reach an object up high)?	0.00%	0.98
Does the child get along well with other children most of the time?	0.04%	0.98
Does the child share things (e.g., food, toys) with others?	0.04%	0.98
Does the child show affection toward others (e.g., hugging parents, brothers, or sisters)?	0.08%	0.98
Does the child wake up frequently at night? ^b^	0.12%	0.96
^a^Is the child frequently sad or upset? ^b^	0.00%	0.95
^a^Does the child have trouble falling asleep on his/her own? ^b^	0.00%	0.94
^a^Does the child stop immediately when told “no” or “stop that?”	0.08%	0.92
^a^Is the child frequently irritable or fussy? ^b^	0.24%	0.86
^a^Does the child show sympathy or look concerned when others are hurt or sad?	3.59%	0.85
^a^Does the child act differently (e.g., shy or afraid) with strangers than he/she does with you and other familiar people?	0.24%	0.85
^a^Is the child able to focus on one task (e.g., playing with friends, eating meal) for more than a few minutes, ignoring other things around him/her?	0.20%	0.82
^a^Does the child follow rules and obey adults?	0.36%	0.82
^a^Does the child have trouble sitting still when asked to by an adult (e.g., for two minutes)? ^b^	0.20%	0.74
^a^Does the child become very upset by loud sounds (e.g., a loud bang, scream)? ^b^	0.40%	0.62
^a^Does the child demand you to be with him/her constantly? ^b^	0.00%	0.62
^a^Is the child able to do two things at the same time (e.g., play a game and listen to you)?	0.81%	0.61
^a^Does the child greet neighbors or other people he/she knows without being told (e.g., by saying hello or gesturing hello)?	0.12%	0.54
^a^When the child is upset, is he/she able to calm down by him/herself?	0.08%	0.53
^a^Does the child put objects or toys back where they belong after using them?	0.36%	0.49
^a^Is the child sometimes impatient or unwilling to wait or hold still when you ask him/her to? ^b^	0.16%	0.44
^a^Does the child act impulsively or without thinking? ^b^	2.46%	0.41
^a^Does the child sometimes save things like candy or new toys for the future?	0.32%	0.38
^a^Does the ever child kick, bite, or hit other children or adults? ^b^	0.08%	0.31
^a^Does the child cry or complain when he/she is made to wait for something he/she wants (e.g., toy, food)? ^b^	0.08%	0.18
*Mean, all items (n = 70)*	*1.40%*	*0.76*
*Mean, final items (n = 44)*	*1.98%*	*0.64*

^a^indicates item that was included in the final reliability and validity analyses

^b^indicates item that was reverse coded

## Abbreviations

BOI: Behavior Observation Inventory
BSID-III: Bayley Scales of Infant Development (III)
ECD: Early Childhood Development
CREDI: Caregiver-Reported Early Development Index

## Footnotes

1 Although not representative of the core ECD domains, one item—whether the child was frequently too sick to play—was borrowed from the MICS ECDI and included alongside the motor items to test its utility in the <3 age group. This health item was tested in the qualitative and quantitative pilots but not included in reliability or validity analyses of the total CREDI scale.

2 In several cases, items appropriate for younger ages (down to 12 months) were included to assess their suitability for older children living in an under-studied, at-risk sample.

3 In the case of items of child behavior traditionally measured using Likert response scales (e.g., never/sometimes/often/always), we integrated “frequency anchors” into the questions themselves to indicate the prevalence of behavior necessary to achieve a “yes” versus a “no” response (e.g., “Does the child get along well with other children *most of the time*?”). These frequency anchors were selected to discriminate between adaptive vs. non-adaptive behavior for each item.
